# Supplementary material for: Room-temperature hyperpolarization of polycrystalline samples with optically polarized triplet electrons: pentacene or nitrogen-vacancy center in diamond?
Source: Magn Reson (Gott). 2021 Feb 17;2(1):33–48. doi: 10.5194/mr-2-33-2021 (PMC10539752; doi:10.5194/mr-2-33-2021)
Supplement: The supplement related to this article is available online at: https://doi.org/10.5194/mr-2-33-2021-supplement. [file mr-2-33-supplement.pdf]

Supplement of Magn. Reson., 2, 33–48, 2021
https://doi.org/10.5194/mr-2-33-2021-supplement
© Author(s) 2021. This work is distributed under
the Creative Commons Attribution 4.0 License.

Open Access
MAGNETIC RESONANCE

Supplement of

Room-temperature hyperpolarization of polycrystalline samples with optically polarized triplet electrons: pentacene or nitrogen-vacancy center in diamond?

Koichiro Miyanishi et al.

Correspondence to: Koichiro Miyanishi (miyanishi@qi.mp.es.osaka-u.ac.jp), Takuya F. Segawa (segawat@ethz.ch), and Makoto Negoro (negoro@qiqb.otri.osaka-u.ac.jp)

The copyright of individual parts of the supplement might differ from the CC BY 4.0 License.

S1 Estimation of the electron spin polarization of the NV$^-$ centers in microdiamonds

Using the EPR spectrum of the nanodiamonds with and without laser irradiation, we estimated the initial electron polarization $P_e^{\rm NV}$ of the NV$^-$ centers between the $m_S = 0$ and the $m_S = -1$ states right after laser irradiation in the magnetic field sweep range. The obtained spectra are shown in Fig. S1. By comparing the signal intensity of the NV$^-$ with and without laser irradiation within the shaded area, which is the optimized range used for the magnetic-field sweep, we obtained an enhancement of ca. 170. The estimated uncertainty, stemming from the noise in the thermal spectrum is $\pm$ 20 %. Having a thermal polarization of 0.095 %, the electron spin polarization of the NV$^-$ right after laser irradiation is estimated to be 16 %. The thermally-polarized spectrum was averaged over 9 h, while the optically-polarized spectrum was recorded in 1 h. Spectra were recorded using a field-swept ESE sequence (Fig. 3(a) in the main text) with an echo delay time of 2.1 μs at a MW frequency of 11.6 GHz. Please note that the sample orientation of the microdiamonds is different to that used in the DNP measurements.

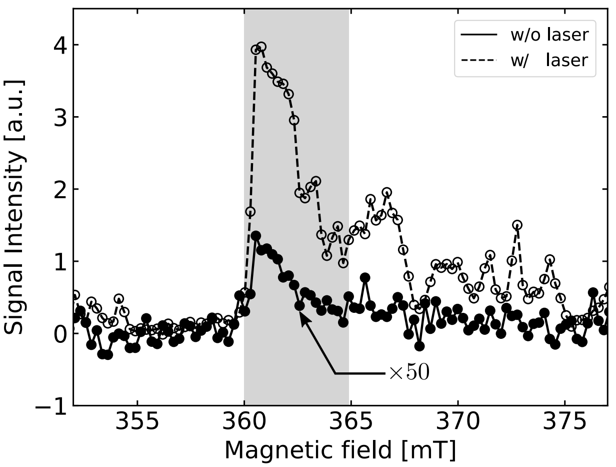

Figure S1. EPR powder spectrum of the microdiamond sample. The dashed line shows the EPR signal of the NV$^-$ centers just after laser irradiation and the solid line shows the EPR signal without laser irradiation. The gray area corresponds to the magnetic field sweep range used for the DNP experiments.

S2 NMR linewidth of ^{13}C in PBA with and without ^{1}H decoupling

The ^{13}C NMR of PBA with and without ^{1}H decoupling is shown in Fig. S2. Both signals are normalized by the maximum signal intensity. The linewidth of the ^{13}C NMR signal with ^{1}H decoupling was 1.2 kHz and that without ^{1}H decoupling was 5.2 kHz. This signal broadening is due to the dipole–dipole interaction between ^{13}C spins and ^{1}H spins, the interaction, which leads to the low spin diffusion coefficient in PBA.

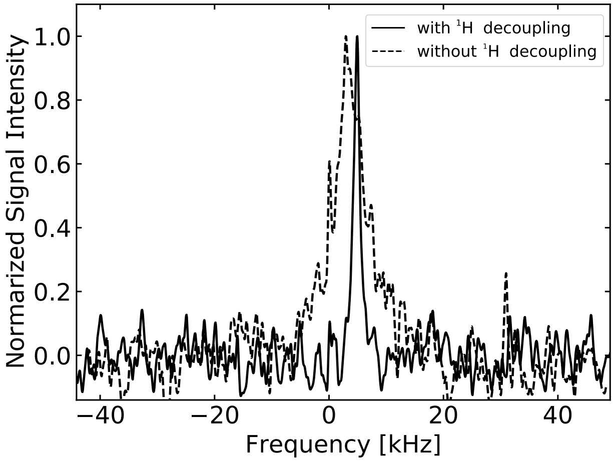

Figure S2. NMR spectra of ^{13}C in PBA. The solid line shows the ^{13}C NMR signals with ^{1}H decoupling and the dashed line without ^{1}H decoupling.

S3 ^{13}C hyperpolarization in microdiamonds at the 'high-field' horn

Hyperpolarized ^{13}C NMR is performed at a magnetic field of 0.46 T (corresponding to the 'high-field' horn of the EPR powder spectrum of the microdiamonds in Fig. 3(d)) at a Larmor frequency of $\omega_{0,\mathrm{C}} = 4.95$ MHz and a DNP-enhanced ^{13}C NMR spectrum is shown in Fig. S3(a). Buildup curves of ^{13}C polarization with laser repetition frequency of $R = 30, 50, 70$ and 100 Hz are shown in Fig. S3(b).

The curve of longitudinal relaxation of the ^{13}C spins in microdiamonds are shown in Fig. S3(c) and the longitudinal relaxation time is $T_{1,\mathrm{C}}^{\mathrm{NV}} = 85 \pm 5$ s. In the longitudinal relaxation measurement, we change the delay time between the DNP process and the NMR measurement.

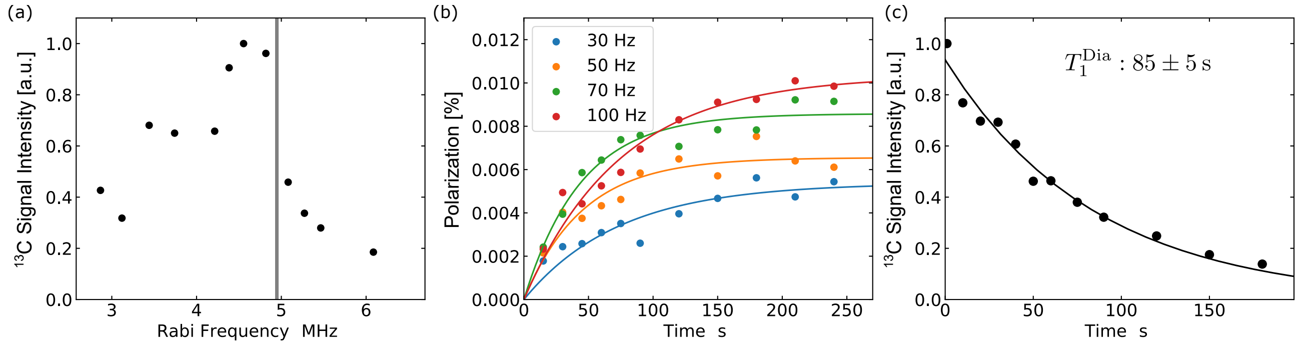

Figure S3. Optical hyperpolarization in microdiamonds at a magnetic field of 0.46 T (corresponding to the 'high-field' horn of the EPR powder spectrum). (a) Signal intensity dependence on the microwave power $\omega_{\mathrm{rabi}}^{\mathrm{MW}}$ at the high-field horn. The gray line shows the ^{13}C Larmor frequency at high-field horn. (b) Buildup curves of ^{13}C polarization in microdiamonds. The blue, orange, green and red denote the laser repetition frequency of 30 Hz, 50 Hz, 70 Hz and 100 Hz. (c) The relaxation curve for the ^{13}C NMR signal of microdiamonds.

S4 Sample pictures

The pictures of the three samples in the glass tubes are shown in Fig. S4.

Fig. S5 shows the effect of oxidation in air on the color of the nanodiamond powders. Both samples are electron irradiated and annealed (800°C in vacuum) nanodiamonds. Fig. S5(a) was taken before oxidation in air, while Fig. S5(b) was taken after oxidation in air. The oxidation procedure removes sp^2 carbon on the surface of the nanodiamonds, which was created during the annealing step. The change in color from grey to white is a chemical key step to perform optical excitation of NV centers in nanodiamonds.

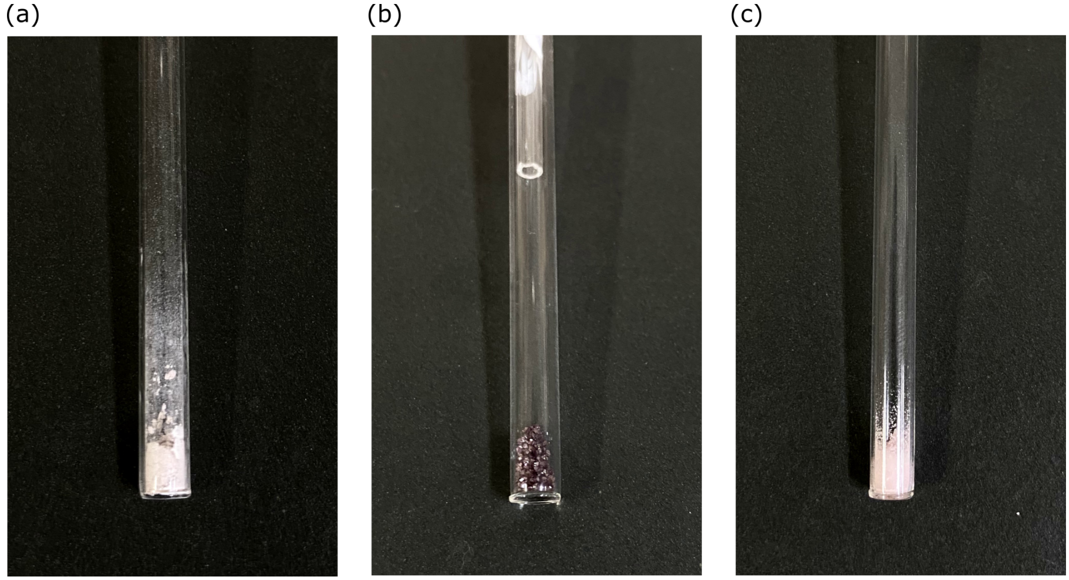

Figure S4. Pictures of the three samples (a) nanodiamonds, (b) microdiamonds and (c) pentacene doped benzoic acid.

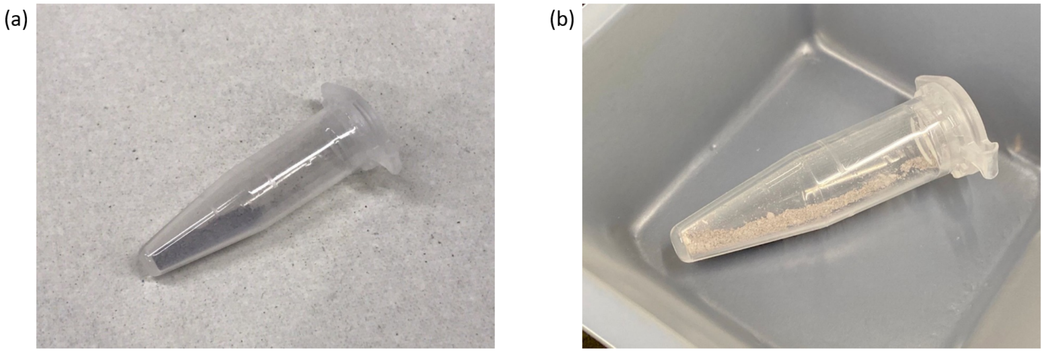

Figure S5. Pictures of electron irradiated and annealed nanodiamonds (a) before oxidation in air (“dark”) and (b) after oxidation in air (“white”).
